# Clinical Efficacy of Prodom-Assisted Urokinase in the Treatment of Male Infertility Caused by Impaired Semen Liquefaction

**DOI:** 10.1155/2021/8862282

**Published:** 2021-01-19

**Authors:** Kaiyi Mao, Zongping Chen, Mengzhi Li, Chengren Gou, Zidong Zhou, Yong Yan, Chao Chen, Tong Liu, Chenghong Zou, Yuhong Yao, Xu Li

**Affiliations:** ^1^Department of Urology, Affiliated Hospital of Zunyi Medical University, Zunyi, Guizhou 563000, China; ^2^Department of Urology, Beijing Shijitan Hospital, Capital Medical University, Beijing 100038, China; ^3^Department of Urology, The Second Affiliated Hospital of Zunyi Medical University, Zunyi, Guizhou 563000, China

## Abstract

**Purpose:**

To evaluate the clinical efficacy of prodom in the administration of urokinase in the vagina in couples with impaired semen liquefaction.

**Materials and Methods:**

Overall, 261 patients with impaired semen liquefaction were randomly divided into prodom-assisted urokinase treatment (PAUT) group (*n* = 91), syringe-assisted urokinase treatment (SAUT) group (*n* = 86), and traditional treatment (TT) group (*n* = 84) in the first stage. If the first stage of treatment failed, other treatment methods were initiated instead and the patients were grouped according to the newer treatment method in the second stage. The pregnancy rate, time-to-conception, and treatment costs were evaluated in each group.

**Results:**

In the first stage, the pregnancy rate in the PAUT, SAUT, and TT groups was 69.23%, 29.07%, and 22.62%, respectively; the time-to-conception was 2.66 ± 1.44, 3.69 ± 2.61, and 3.86 ± 3.00 months, respectively; the treatment costs were 658.18 ± 398.40, 666.67 ± 507.50, and 680.56 ± 480.94 $, respectively. The pregnancy rate and time-to-conception were different in the PAUT group compared with those in SAUT and TT groups (all *P* < 0.05). However, the difference in treatment costs was not significant (*P* = 0.717). In the second stage, 154 nonpregnant patients were divided into nine treatment groups, and the effects of changing TT to PAUT on the pregnancy rate, time-to-conception, and treatment costs were observed to be different from those of other treatments (all *P* < 0.05).

**Conclusion:**

Prodom-assisted urokinase can effectively treat male infertility secondary to impaired semen liquefaction.

## 1. Introduction

Impaired semen liquefaction (ISL) refers to the requirement of more than 1 h for semen liquefaction after ejaculation [1]. It is one of the commonest causes of male infertility and accounts for infertility in approximately 2.51%–42.65% of males [2–5]. Currently, the pathogenesis of ISL is poorly understood. Several studies have suggested that ISL is caused by a decrease in plasminogen activators in the seminal plasma, such as urokinase and chymotrypsin, and their receptors [6–13]. Western and Chinese medicines are the primary methods used to treat ISL [4, 5, 14–22]. However, because of the complex metabolic mechanisms in the human body, neither oral Western nor Chinese medicines can resolve ISL in *in vivo* approaches [4, 5]. Therefore, the focus of treatment has shifted toward improving ISL using *in vitro* approaches to identify an ideal therapeutic approach [14–19].

Of them, chymotrypsin and urokinase have been reported to improve ISL, method of using them varies, and, hence, the outcomes vary [14, 16–20]. We previously used chymotrypsin to treat male infertility secondary to ISL using a special device—called “prodom”—that we designed, which yielded encouraging results [19]. Currently, prodom-assisted urokinase therapy in ISL has not been reported in clinical practice. Therefore, the purpose of this study was to evaluate the clinical efficacy of prodom in treating ISL-induced male infertility using urokinase therapy.

## 2. Materials and Methods

### 2.1. Patients

In this randomized controlled trial study, consecutive data of 313 patients with ISL from January 1, 2012, to December 31, 2019, were collected from the Affiliated Hospital of Zunyi Medical University, China. The inclusion criteria were the following: complete clinical records (outpatient from January 1, 2012, to December 31, 2019) with follow-up of 9-96 months. The exclusion criteria were the following: (1) unimpaired semen liquefaction; (2) organic diseases, including uremia, cirrhosis and liver failure, lung failure, endocrine dysfunction, mental disorders, azoospermia, and severe oligospermia; (3) incomplete clinical records; or (4) termination of the treatment or refusal for follow-up visits. While 52 patients terminated the treatment prematurely, the remaining 261 completed the study. The Institutional Review Board of the Affiliated Hospital of Zunyi Medical University approved the study in January 2012, 2015 and 2018.

### 2.2. Experimental Group and Measures in Each Group

**Figure 1:**
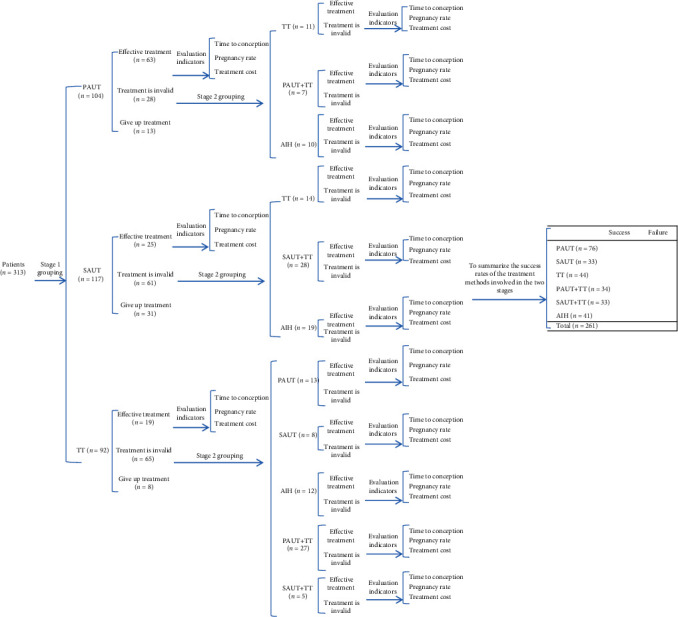
Flow chart of the experimental design.

The 261 patients with ISL were divided into prodom-assisted urokinase treatment (PAUT) group (*n* = 91), syringe-assisted urokinase treatment (SAUT) group (*n* = 86), and traditional treatment (TT) group (*n* = 84). Randomization included assigning a number to each patient using the random number table according to the order of visit. The number was then divided by 3, and patients with remainders of 0, 1, and 2 were assigned to the PAUT, SAUT, and TT group, respectively. The allocation was concealed using sealed envelopes to randomize the distribution. In each of the groups, pregnancy, time-to-conception, and treatment costs were evaluated in the first stage. If the first stage of treatment failed, other treatment methods were initiated instead and the patients were grouped according to the newer treatment method in the second stage. Conception and live birth rates were used as measures of treatment success, and the success and failure rates were calculated for each treatment as well as all treatments cumulatively (see Figure 1). Patients in the PAUT group were administered urokinase (Tianjin Biochemical Pharmaceutical Co. Ltd., China, batch No. 031802014). One milliliter of 10,000 IU urokinase was injected into the vagina of the partner through the prodom during intercourse, which was synchronously released with ejaculation. This helped urokinase to be mixed with the semen. In the SAUT group, 0.5 mL of urokinase (5,000 IU) was injected into the vagina using a 5-milliliter syringe before the penis was inserted as well as after the penis was removed during intercourse. In the TT group, 1,000 mg of vitamin C and 100 mg of vitamin E were administered everyday along with 10 mL zinc gluconate and a spermatogenic tablet (containing a traditional Chinese medicine) administered thrice a day as supplements.

### 2.3. Prodom: Composition and Operational Process

Prodom is a type of auxiliary reproductive device that temporarily sticks on the penis that aids the male partner in injecting a drug into the vagina of female partner simultaneous with ejaculation such that the drug can be mixed with the semen in the vagina, thus improving the semen composition and contributing toward conception. The prodom described in this study was primarily composed of a polyurethane (PU) film and an injection catheter. It was coated with pressure-sensitive adhesive on the inner side of the PU film [19]. Operating process was as follows: (1) before coitus, 1 mL of sterile saline and urokinase solution was injected with a syringe in the vagina, while the prodom was pasted onto the erect penis using the pressure-sensitive adhesive set on the PU film. (2) The urokinase injection through the wife into the vagina was synchronized with ejaculation, thereby blending it with the ejaculation in the vagina [19].

### 2.4. Steps for the Use of Prodom-Assisted Urokinase and Syringe-Assisted Urokinase

The specific steps have been described previously [19]. In this study, chymotrypsin was replaced with urokinase.

### 2.5. Clinical Data Extraction

The available data included semen parameters (volume, pH, liquefaction time, and sperm density, motility, live rate, and morphology), duration of infertility, routine urine tests (leukocytes and red blood cells), prostatic enlargement, seminal vesicle enlargement, white blood cells in prostatic massage fluid, chronic prostatitis, history of chronic prostatitis, history of seminal vesiculitis, age of both partners, pregnancy, time-to-conception, and treatment costs. Semen parameters were measured by semiautomatic semen analyzer (BEION S3-3, BEIONMED®, China).

### 2.6. Diagnosis Standards

ISL refers to a condition in which the liquefaction time exceeds 60 min. Male infertility refers to the failure to conceive despite having regular intercourse without contraception a couple living together for more than 1 year and without female infertility. ISL and male infertility were diagnosed according to the Fifth Edition (2010) of *the WHO laboratory test manual for human semen and sperm-cervical mucus interactions* [1].

### 2.7. Follow-Up

The median follow-up time was 4.8 (range, 0.75–8) years. All the patients were followed up via telephone and regular outpatient visits. The parameters recorded during follow-ups through telephone consultation included the health status, pregnancy status, and time of conception. Outpatient follow-ups included general physical examinations, routine blood and biochemical examinations, and analysis of semen quality.

### 2.8. Statistical Analyses

Statistical analyses were performed using SPSS v18 (SPSS Inc., Chicago, IL, USA). The selected characteristics (including the clinical parameters described above) were compared between the treatment groups using the chi-squared test for categorical variables and *t*-test and one-way analysis of variance (one-way ANOVA) for quantitative data. Numerical variables are presented as mean ± standard deviation, and categorical variables are presented as percentages. *P* < 0.05 was considered significant.

## 3. Results

### 3.1. Baseline Characteristics

The duration since infertility was diagnosed was 1.5–21 years while the duration of infertility was 4.2 ± 2.4 years. The men were of 21–46 years old (mean, 32.5 years), while their partners were 21–37 years old (mean, 27.5 years). Overall, 261 patients were divided into PAUT (*n* = 91), SAUT (*n* = 86), and TT (*n* = 84) groups. There were no significant differences between the groups in terms of semen parameters; duration of infertility; urine findings; prostatic enlargement; seminal vesicle enlargement; white blood cells in prostatic massage fluid, chronic prostatitis, or history of chronic prostatitis; history of seminal vesiculitis; and ages of the patients and their partners (*P* > 0.05) (Table 1). Of the 52 patients who terminated the treatment, the PAUT group accounted for 12.50% (13/104), SAUT group for 26.50% (31/117), and TT group for 8.70% (8/92) of them. The rate of abandoning treatment was lower in the PAUT group than those in the other two groups. The differences between the groups were statistically significant (*P* = 0.001).

### 3.2. Comparison of Pregnancy Rate, Time-to-Conception, and Treatment Costs in the First Stage of Treatment

In the first stage of treatment, in the PAUT, SAUT, and TT groups, As presented in Table 2, the pregnancy rate was 69.23% (63/91), 29.07% (25/86), and 22.62% (19/84), respectively; time-to-conception was 2.66 ± 1.44 months, 3.69 ± 2.61 months, and 3.86 ± 3.00 months, respectively; cost of treatment was 658.18 ± 398.40, 666.67 ± 507.50, and 680.56 ± 480.94 $, respectively. The three treatment methods were not similar in terms of the pregnancy rate (*χ*^2^ = 256.075 and *P* < 0.0001) and time-to-conception (*F* = 20.411 and *P* < 0.0001); however, the difference in treatment cost (*F* = 0.333 and *P* =0.717) was not significant. Of them, the effects of PAUT on pregnancy rate and time-to-conception were different than those of SAUT and TT (*P* < 0.05). Therefore, it can be considered that PAUT is superior to SAUT and TT in increasing the pregnancy rate and shortening the time-to-conception.

### 3.3. Pregnancy Rate, Time-to-Conception, and Treatment Costs between Groups in the Second Stage of Treatment

After 9 months, 154 patients who were unable to achieve pregnancy in the first stage of treatment in PAUT (*n* = 28), SAUT (*n* = 61), and TT groups (*n* = 65) were assigned to receive other treatments in the second stage. As presented in Table 3, the pregnancy rate was 36.36% (4/11), time-to-conception was 19.50 ± 5.84 months, and cost of treatment was $ 2,538 ± 179 in the patients in PAUT group who were switched to TT group. The pregnancy rate was 50% (17/34), time-to-conception was 17.75 ± 6.02 months, and cost of treatment was $ 4,464 ± 177 in the PAUT group combined with the TT group. The pregnancy rate was 70% (7/10), time-to-conception was 21.40 ± 16.73 months, and cost of treatment was $ 19,621 ± 6,164 in the patients in PAUT group who underwent artificial insemination with husband's semen (AIH). The pregnancy rate was 42.11% (8/19), time-to-conception was 17.86 ± 5.91 months, and cost of treatment was $ 2,597 ± 223 in the patients in SAUT group who were switched to TT group. The pregnancy rate was 51.52% (17/33), time-to-conception was 13.83 ± 7.21 months, and cost of treatment was $ 2,248 ± 706 in the SAUT group combined with the TT group. The pregnancy rate was 42.11% (8/19), time-to-conception was 19.84 ± 15.90 months, and cost of treatment was $ 18,586 ± 4,237 in the patients in SAUT group who underwent AIH. The pregnancy rate was 69.23% (9/13), time-to-conception was 12.67 ± 4.40 months, and cost of treatment was $ 1,358 ± 266 in the patients in TT group who were switched to PAUT group. The pregnancy rate was 75.00% (6/8), time-to-conception was 16.62 ± 4.70 months, and cost of treatment was $ 2,129 ± 262 in the patients in TT group who were switched to SAUT group. The pregnancy rate was 66.67% (8/12), time-to-conception was 21.83 ± 18.75 months, and cost of treatment was $ 20,726 ± 3,336 in the patients in TT group who underwent AIH. The effects on pregnancy rate (*χ*^2^ = 8.405 and *P* = 0.015), time-to-conception (*F* = 39.876 and *P* < 0.0001), and cost of treatment (*F* = 129.567 and *P* < 0.0001) of the nine treatments mentioned above were not similar to each other. Furthermore, in these treatment groups, the effects of switching from TT to PAUT on the pregnancy rate, time-to-conception, and cost of treatment were different from those of the other treatments (all *P* < 0.05). Therefore, our results demonstrated that switching from TT to PAUT was superior to the other treatments in the pregnancy rate, time-to-conception, and cost of treatment.

### 3.4. Analysis of the Success Rates of Different Treatments

We summarized the treatment methods, including PAUT, SAUT, TT, PAUT+TT, SAUT+TT, and AIH. We used the conception and live birth rates as measures of the treatment success and calculated the success and failure rates for each treatment as well as the overall success and failure rates for all the treatments. As presented in Table 4, we sorted the statistics of the efficacies of these six treatment methods. Of them, PAUT had a 94.74% (72/76) success rate and 5.26% (4/76) failure rate; SAUT had 87.88% (29/33) success rate and 12.12% (4/33) failure rate; TT had 65.91% (29/44) success rate and 34.09% (15/44) failure rate; PAUT+TT had success and failure rates of 50.00% (17/34) each; SAUT+TT had 51.52% (17/33) success rate and 48.48% (16/33) failure rate; AIH had 56.10% (23/41) success rate and 43.09% (18/41) failure rate. The overall treatment success rate was 71.65% (187/261), and the failure rate was 28.35% (74/261). Chi-square check was used for Row x List, and the success rates of the above six treatments were not the same (*χ*^2^ = 38.213 and *P* < 0.0001). Furthermore, of them, PAUT was different from other groups in pairwise comparisons (all *P* < 0.05). These results indicate that the success rate of PAUT was higher than those of the other treatments.

## 4. Discussion

Currently, there are several methods to treat ISL, including oral administration, muscle injection, transurethral administration, transrectal administration, transvaginal administration, and acupuncture. All these methods have been reported to be effective although they have their own limitations; consequently, satisfactory results are not achieved in some patients. Previous studies have confirmed that the addition of chymotrypsin to the semen after ejaculation could completely correct ISL and improve sperm vitality without damaging the sperm quality. We used a syringe/prodom to assist the injection of chymotrypsin into the vagina during coitus to treat ISL, which produced better results. Further, it was previously reported that the effects of treatment using prodom-assisted chymotrypsin in the vagina were better than those of using a syringe to inject chymotrypsin in the vagina [19]. However, PAUT has not been reported previously.

In this study, we evaluated the clinical efficacy of prodom-assisted urokinase in treating ISL. The results demonstrated that the pregnancy rate was 69.23% in the PAUT group, which was higher than those of both the SAUT (29.07%) and TT (22.62%) groups. Time-to-conception was 2.66 ± 1.44 months, which was significantly lower than those in both the SAUT (3.69 ± 2.61 months) and TT (3.86 ± 3.00 months) groups in the first stage (all *P* < 0.05). However, the cost of treatment in the PAUT, SAUT, and TT groups was 658.18 ± 398.40, 666.67 ± 507.50, and 680.56 ± 480.94 $, respectively, which was not significantly different (*P* = 0.717). However, in the second stage, after switching from TT to PAUT, the effects on the pregnancy rate, time-to-conception, and cost of treatment were significantly different from those of the other treatments (all *P* < 0.05). Consequently, these results prove that PAUT is superior to other treatments in increasing the pregnancy rate, shortening the time-to-conception, and reducing the cost of treatment.

According to the results of this study, in the first stage of treatment, PAUT was superior to SAUT and TT in increasing the pregnancy rate and shortening the time-to-conception (Table 2). In the second stage of treatment, changing TT to PAUT was superior to the other treatments in increasing the pregnancy rate, shortening the time-to-conception, and reducing the cost of treatment (Table 3).

Regarding the overall efficacy and specific efficacy of different treatments used in ISL, we summarized the six treatment methods of PAUT, SAUT, TT, PAUT+TT, SAUT+TT, and AIH. The overall treatment success rate was 71.65%, and the treatment success rate of PAUT, SAUT, and TT was 94.74%, 87.88%, and 65.91%, respectively (Table 4). The treatment success rates of PAUT and SAUT were higher than that of TT and the overall treatment success rate. Additionally, the specific success rates of these six treatment regimens were not similar (*χ*^2^ = 38.213 and *P* < 0.0001). Of them, PAUT was different from other groups in pairwise comparisons (all *P* < 0.05). Therefore, the results prove that the efficacy of PAUT was higher than those of the other treatment regimens.

**Figure 2:**
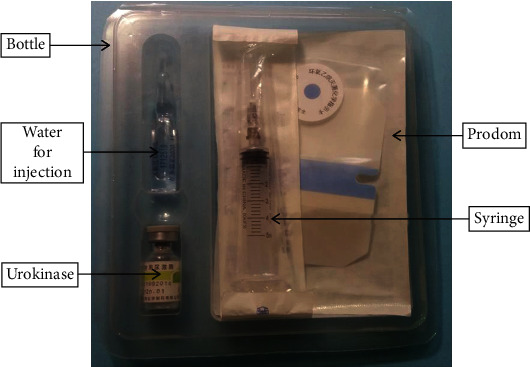
Packing sample product label of prodom-assisted urokinase.

Previous studies have reported that the pregnancy rate obtained by treating ISL using Chinese medicines was 38.71% [4], while that obtained with the use of chymotrypsin was 21%–53.21% [18, 19]. The pregnancy rate reported was 69.23% using PAUT in this study, which is higher than those in previous reports. Furthermore, in terms of time-to-conception and cost of treatment, PAUT was also reported to be superior to other treatments in this study and those in previous reports. Additionally, complications, such as allergies, genital tract injuries, bleeding, and infections, were not observed with this treatment regimen. Therefore, we recommend the use of PAUT in treating ISL (Figure 2).

However, the present study had several limitations. The overall pregnancy rate was 71.65% (187/261), which implied that 28.35% (74/261) of patients did not benefit from the treatment regimens used in this study. Therefore, new treatment methods and treatment policies should be further explored [21–25]. We are working toward transferring the patients with response failure for the first two stages of treatment to the third stage of treatment and use a rotation model with the aforementioned treatments to improve the outcomes. Furthermore, this study was conducted only in the Chinese population, which included a majority of Han Chinese population but only a small proportion of Hui, Miao, Buyi, and Gelao populations who have different lifestyles. Our study did not subdivide the participants based on such populations. Additionally, this study included a relatively small number of patients from a single center. These limitations may have affected the results and conclusions. Therefore, larger and multicenter studies are necessary to corroborate our findings.

## 5. Conclusion

In conclusion, the prodom can increase the rate of pregnancy, shorten the time-to-conception, and reduce cost of treatment in patients with ISL by the virtue of assisting in the administration of urokinase into the vagina.

## Figures and Tables

**Table 1:** Baseline characteristics of clinical data in each group.

Groups parameters	PAUT group (*n* = 91)	SAUT group (*n* = 86)	TT group (*n* = 84)	*P* value
Male's age (year)	30.4 ± 6.3	31.9 ± 6.3	30.1 ± 5.5	0.103^†^
Female's age (year)	27.3 ± 4.9	28.6 ± 4.6	27.2 ± 4.5	0.097^†^
Semen parameters				
Emen volume (mL)	3.46 ± 0.89	3.41 ± 0.84	3.49 ± 0.78	0.821^†^
pH	7.40 ± 0.16	7.44 ± 0.17	7.39 ± 0.15	0.084^†^
Liquefaction time (min)	>60	>60	>60	NS
Sperm density (×10^6^/mL)	45.89 ± 10.58	45.35 ± 9.28	45.35 ± 9.13	0.912^†^
Sperm motility, %				
a class	18.89 ± 3.29	19.17 ± 3.64	18.69 ± 3.31	0.650^†^
a+b class	42.45 ± 5.52	42.31 ± 4.94	42.94 ± 5.71	0.729^†^
Live sperm rate, %	56.27 ± 8.88	56.05 ± 9.06	55.33 ± 8.85	0.771^†^
Sperm morphology, %				
Normal	29.84 ± 7.37	29.77 ± 7.44	30.00 ± 6.94	0.977^†^
Abnormal	70.16 ± 7.37	70.23 ± 7.44	70.00 ± 6.94	0.977^†^
Duration time of infertility (year)	4.78 ± 3.63	4.78 ± 2.89	4.45 ± 2.60	0.725^†^
Routine urine test				
WBC, %				0.611^∗^
(+)	5.49 (5/91)	7.14 (6/84)	7.41 (6/81)	
(-)	94.51 (86/91)	92.86 (78/84)	92.59 (75/81)	
RBC, %				0.781^∗^
(+)	5.49 (5/91)	5.95 (5/84)	7.41 (6/81)	
(-)	94.51 (86/91)	94.05 (79/84)	92.59 (75/81)	
Prostatic enlargement, %				0.938^∗^
Yes	9.89 (9/91)	7.14 (6/84)	8.64 (7/81)	
No	90.11 (82/91)	92.86 (78/84)	91.36 (74/81)	
Seminal vesicle enlargement, %				0.760^∗^
Yes	5.49 (5/91)	4.76 (4/84)	8.64 (7/81)	
No	94.51 (86/91)	95.24 (80/84)	91.36 (74/81)	
Prostatic massage fluid, %				0.798^∗^
WBC (+)	6.59 (6/91)	7.14 (6/84)	8.64 (7/81)	
WBC (-)	93.41 (85/91)	92.86 (78/84)	91.36 (74/81)	
Chronic prostatitis, %				0.688^∗^
Yes	12.09 (11/91)	7.14 (6/84)	8.64 (7/81)	
No	87.91 (80/91)	92.86 (78/84)	91.36 (74/81)	
History of chronic prostatitis, %				0.577^∗^
Yes	4.40 (4/91)	8.33 (7/84)	7.41 (6/81)	
No	95.60 (87/91)	91.67 (77/84)	92.59 (75/81)	
History of seminal vesiculitis, %				0.993^∗^
Yes	4.40 (4/91)	4.76 (4/84)	4.94 (4/81)	
No	95.60 (87/91)	95.24 (80/84)	95.06 (77/81)	
Give up treatment, %	12.50 (13/104)	26.50 (31/117)	8.70 (8/92)	**0.001** ^∗^

Abbreviations: PAUT: prodom-assisted urokinase treatment; SAUT: syringe-assisted urokinase treatment; TT: traditional treatment. *P* values were calculated using ^†^one-way analysis of variance (one-way ANOVA) and ^∗^chi-squared; boldface text represents statistical significance.

**Table 2:** Pregnancy rate/time to conception/treatment cost between groups were compared in the first stage.

Groups	PAUT group (*n*=91)	SAUT group (*n*=86)	TT group (*n*=84)	Total (*n*=261)	*x* ^2^ */F*	*P* value
Parameters						
Pregnancy rate, %	69.23 (63/91)	29.07 (25/86)	22.62 (19/84)	41.00 (107/261)	256.075	**<0.0001** ^∗^
Time to conception (months)	1.44±2.66	3.69±2.61	3.86±3.00	3.02±2.96	20.411	**<0.0001** ^†^
Treatment cost ($)	658.18±398.40	666.67±507.50	680.56±480.94	692.56±416.03	0.333	0.717^†^

Abbreviations: PAUT: prodom-assisted urokinase treatment; SAUT: syringe-assisted urokinase treatment; TT: traditional treatment. *P* values were calculated using ^∗^chi-squared and ^†^one-way analysis of variance (one-way ANOVA). In the first stage of treatment, the three treatment methods were not the same in terms of the influence of pregnancy rate (*χ*^2^=256.075 and *P* < 0.0001) and time to conception (*F*=20.411 and *P* < 0.0001), and the difference in treatment cost (*F*=0.333 and *P* = 0.717) was not significant. Among them, the effect of PAUT on pregnancy rate and time to conception was different from that of SAUT and TT (all *P* < 0.05). It can be considered that the PAUT is superior to the SAUT and the TT in increasing the pregnancy rate and shortening the time to conception. The boldface represents statistical significance.

**Table 3:** Pregnancy rate/time to conception/treatment cost between groups were compared in the second stage.

Groups	PAUT was changed to TT group (*n*=11)	PAUT combined with TT group (*n*=34)	PAUT was changed to AIH (*n*=10)	SAUT was changed to TT group (*n*=14)	SAUT combined with TT group (*n*=33)	SAUT was changed to AIH (*n*=19)	TT was changed to PAUT group (*n*=13)	TT was changed to SAUT group (*n*=8)	TT was changed to AIH (*n*=12)	Total (*n*=154)	*F*	*P* value
Parameters												
Pregnancy rate (%)	36.36 (4/11)	50.00 (17/34)	70.00 (7/10)	42.86 (6/14)	51.52 (17/33)	42.11 (8/19)	69.23 (9/13)	75.00(6/8)	66.67 (8/12)	49.35 (76/154)	8.405	**0.015**
Time to conception (months)	19.50±5.84	17.75±6.02	21.40±16.73	17.86±5.91	13.83±7.21	19.84±15.90	12.67±4.40	16.62±4.70	21.83±18.75	17.75±11.20	39.876	**<0.0001**
Treatment cost ($)	2,538±179	4,464±177	19,621±6,164	2,597±223	2,248±706	18,586±4,237	1,358±266	2,129±262	20,726±3,336	7,837±6,864	129.567	**<0.0001**

Abbreviations: PAUT: prodom-assisted urokinase treatment; SAUT: syringe-assisted urokinase treatment; TT: traditional treatment; AIH: artificial insemination with husband's semen. *P* values were calculated using one-way analysis of variance (one-way ANOVA). In the second stage of treatment, the nine treatment methods were not the same in terms of the influence of pregnancy rate (*χ*^2^=8.405 and *P* = 0.015), time to conception (*F*=39.876 and *P*<0.0001), and the difference in treatment cost (*F*=129.567 and *P* < 0.0001). And, among them, the effects of TT changed to PAUT on the pregnancy rate, the time to conception, and the treatment cost were different from that of the other treatments (all *P* < 0.05). It can be considered that the TT changed to PAUT is superior to the other treatments in increasing the pregnancy rate, shortening the time to conception, and reducing the cost of treatment. The boldface represents statistical significance.

**Table 4:** Compare the efficacy of different treatments in this study.

Treatments	Therapeutic efficiency		Therapeutic inefficiency		Total
	*n*1	%	*n*2	%	
PAUT	72	94.74 (72/76)*^Δ Φ Σ^*	4	5.26 (4/76)	76
SAUT	29	87.88 (29/33)*^Δ Φ Σ^*	4	12.12 (4/33)	33
TT	29	65.91 (29/44)*^Δ^*	15	34.09 (15/44)	44
PAUT+TT	17	50.00 (17/34)*^Δ^*	17	50.00 (17/34)	34
SAUT+TT	17	51.52 (17/33)*^Δ^*	16	48.48 (16/33)	33
AIH	23	56.10 (23/41)*^Δ^*	18	43.90 (18/41)	41

Total	187	71.65 (187/261)	74	28.35 (74/261)	261

Abbreviations: PAUT: prodom-assisted urokinase treatment; SAUT: syringe-assisted urokinase treatment; TT: traditional treatment; AIH: artificial insemination with husband's semen. The chi-square check is used for Row x List, and the efficacy of the above six treatment regimens is not all the same (*χ*^2^=38.213 and *^Δ^P* < 0.0001). Among them, there was no significant difference in the efficacy of PUAT and SAUT (*χ*^2^=0.270 and *^Φ^P* = 0.550), but there was difference between them and other treatments (all *^Σ^P* < 0.05). It can be considered that the efficacy of PAUT and SAUT was higher than that of the other treatments.

## Data Availability

The data used to support the findings of this study are included within the article.
